# The Gastroprotective Effect of Small Molecule Oligopeptides Isolated from Walnut (*Juglans regia* L.) against Ethanol-Induced Gastric Mucosal Injury in Rats

**DOI:** 10.3390/nu12041138

**Published:** 2020-04-18

**Authors:** Rui Liu, Yun-Tao Hao, Na Zhu, Xin-Ran Liu, Jia-Wei Kang, Rui-Xue Mao, Chao Hou, Yong Li

**Affiliations:** Department of Nutrition and Food Hygiene, School of Public Health, Peking University, Beijing 100191, China; lrui_pku@163.com (R.L.); haoyuntaolly@163.com (Y.-T.H.); summer920503@163.com (N.Z.); liuhappy07@163.com (X.-R.L.); kjiawei@yeah.net (J.-W.K.); maoruixue@163.com (R.-X.M.); houchao_HC@163.com (C.H.)

**Keywords:** walnut oligopeptides, gastroprotective, inflammatory cytokines, oxidative stress, apoptosis

## Abstract

The study investigated the protective effect of walnut oligopeptides (WOPs) against ethanol-induced gastric injury using Sprague-Dawley (SD) rats. Rats were randomly divided into seven groups based on body weight (10/group), normal group, ethanol group, whey protein group (220 mg/kg body weight), omeprazole group (20 mg/kg body weight), and three WOPs groups (220, 440, 880 mg/kg body weight). After 30 days of treatment with WOPs, rats were given 5 mL/kg absolute ethanol by gavage to induce gastric mucosal injury. Gastric ulcer index (GUI) were determined and the following measured; gastric content pH, gastric mucin, endogenous pepsinogens (PG), prostaglandin E2 (PGE2), inflammatory cytokines, oxidative stress indicators, and the expression of apoptosis-related proteins were measured to evaluate the gastroprotective effect of WOPs. The results showed that the administration with WOPs markedly mitigated the hemorrhagic gastric lesions caused by ethanol in rats, and decreased the GUI, the gastric content pH, PG1, PG2, and NO levels, enhanced mucin and PGE2. Also, WOPs repressed gastric inflammation through the reduction of TNF-α, IL-6, IL-1β and increase IL-10 levels, and revealed antioxidant properties with the enhancement of superoxide dismutase, glutathione, and catalase activity, while reduction of malondialdehyde. Moreover, WOPs treatment significantly down-regulated Bax, caspase-3 and nuclear factor-κB p65 (NF-κB p65) expression, while up-regulating the expression of Bcl-2 and inhibitor kappa Bα (IκBα) protein. These results indicated that WOPs have protective effects against ethanol-induced gastric mucosal injury in rats through anti-inflammatory, anti-oxidation, and anti-apoptosis mechanisms.

## 1. Introduction

Gastric ulcers, characterized by mucosal damage, are induced by nonsteroidal anti-inflammatory drugs, potassium chloride, alcohol intake, and Helicobacter pylori, etc., [1]. Ethanol is one of the most common gastric mucosal invasion factors. With the development of society and increased consumption of alcohol, the ethanol-induced gastric ulcer has become a prominent gastrointestinal disease [2]. World Health Organization report showed that drinking alcohol caused about 3 million deaths globally in 2016 (about 2.3 million men and about 700,000 women), accounting for 5.3 percent of all deaths. Diseases of the digestive system accounted for 21.3 percent of deaths attributable to alcohol consumption, placing a huge burden on global public health [3]. Experimentally, ethanol penetrates easily and rapidly into the gastric mucosa, then directly induces gastric mucosa lesions primarily manifested as extended hemorrhagic bands, enlarged submucosal edema, and mucosal fragmentation, which in turn reduces the generation of gastric mucosal protective factors and further aggravating gastric mucosal damage [4,5]. Moreover, there is increasing evidence that ethanol mediates various pathological events directly or indirectly via different pathophysiological pathways to form ulcers. Studies have confirmed ethanol can induce gastric mucosa to over synthesize and secrete a variety of inflammatory cytokines and oxygen free radicals, such as tumor necrosis factor α (TNF-α) and reactive oxygen species (ROS). In addition to direct damage to gastric mucosa, these factors are involved in the process of ethanol-induced excessive inflammation, oxidative damage, cell necrosis, and apoptosis of gastric mucosal [6,7]. However, the majority of anti-ulcer drugs, including proton pump inhibitors, antacids, and antihistaminic agents, currently used in the treatment of peptic ulcers showed limited efficacy and multiple adverse side effects [2,4,8]. Therefore, exploring the potential mechanism and finding safe and effective agents for the protection of ethanol-mediated gastric mucosal injury is of great importance.

Currently, bioactive peptides, especially those derived from edible substances, have drawn increasing attention of researchers because of their multiple physiological functions, such as immunomodulatory, inflammatory inhibition, antioxidant, anti-fatigue effects, etc., [9,10,11,12,13]. Several studies have been demonstrated that whey protein isolate [14], collagen hydrolysates [14], wheat peptides [15], and cod (*Gadus macrocephalus*) skin collagen peptides [16], can protect against ethanol-induced gastric damage.

Walnuts (*Juglans regia* L.) are one of the most widespread tree nuts in the world [17]. Studies have demonstrated that walnut contains various functional components including unsaturated fatty acids, dietary fibers, polyphenols, flavones, proteins, and peptides [18,19]. Walnuts have health-promoting effects, such as antifungal, anti-inflammatory and hypotensive properties and antioxidant activities [17,18,19,20,21]. Walnut oligopeptides (WOPs), which are extracted from walnut, characterized by lower molecular weight, more digestible and absorbable properties. Previous studies reported that WOPs has anti-oxidant, anti-inflammation, and anti-fatigue effects in mice [22,23]. However, there is no report on the gastroprotective effect of WOPs. Therefore, we speculated that WOPs could be considered an effective agent to combat gastric mucosal injury induced by ethanol which is related to oxidative stress imbalance, inflammation, and apoptosis. Hence, this study aimed to explore the possible protective effects of WOPs against ethanol-induced gastric mucosal injury and its mechanism in rats.

## 2. Materials and Methods

### 2.1. Preparation of WOPs Sample 

WOPs were extracted from the proteins of walnut (*Juglans regia* L.) via enzymatic hydrolysis and provided by Jilin Taigu Biological Engineering Co., Ltd. (Jilin, China). Briefly, walnut residual proteins were homogenized, centrifugated, and then hydrolyzed by multiple proteases. Then, nanofiltration, cryoconcentration, decolorization, purification, and spray drying were performed to obtain WOPs powders. After being purified by high-performance liquid chromatography (HPLC, Agilent, CA, USA), matrix-assisted laser desorption ionization time-of-flight mass spectrometry (MALDI-TOF-MS, Linear, NV, USA) and automatic amino acid analyzer (Hitachi, Tokyo, Japan) were used to determine the molecular weight distribution and free amino acids amount of WOPs sample separately. The identification results showed that the small molecule oligopeptides with relative molecular weight <1000 Da accounted for 86.5% of WOPs sample. Further analysis found that the free amino acids accounted for 2.98% and the detailed amino acid composition of the sample was described in our previous reports [22,23].

### 2.2. Chemicals and Reagents

Absolute ethanol (ETOH) was purchased from Sinopharm Chemical Reagent Beijing Co., Ltd (Beijing, China). Whey protein was obtained from Jilin Taigu Biological Engineering Co., Ltd. (Jilin, China). Omeprazole was purchased from Hunan Dino Pharmaceutical Limited by Share Ltd (Hunan, China). The serum alanine aminotransferase (ALT), aspartate aminotransferase (AST) assay kits were obtained from Yingkexinchuang Science and Technology Ltd. (Macau, China). The prostaglandin E2 (PGE2), pepsinogens, mucin, superoxide dismutase (SOD), nitric oxide(NO), malondialdehyde (MDA), catalase (CAT), glutathione (GSH), myeloperoxidase (MPO), mucin, tumor necrosis factor α (TNF-α), interleukin (IL)-6, interleukin (IL)-1β, and interleukin (IL)-10 assay kits were purchased from Nanjing Jiancheng Bioengineering Institute (Nanjing, China). The bicinchoninic acid (BCA) protein assay kit and RIPA lysis buffer were purchased from Beyotime Institute of Biotechnology (Beijing, China). The primary antibodies against rabbit nuclear factor-κB p65 (NF-κB p65) and Bax were obtained from Cell Signaling Technology, Inc. (CST); inhibitor kappa Bα (IκBα), Bcl-2, caspase-3, and heat shock protein70 (HSP70) were obtained from Abcam (Cambridge, UK).

### 2.3. Animals and Experimental Design

Sprague-Dawley (SD) rats (male, weighing 180–220 g, 6–8 weeks) in a specific pathogen-free condition were obtained from the Department of Laboratory Animal Science, at Peking University (Laboratory animal production license No.: SCXK (Jing) 2016-0010; Laboratory animal use license No.: SYXK (Jing) 2016-0041). The rats were kept in a rat laboratory in the Department of Laboratory Animal Science, which is in a filter-protected and air-conditioned room with constant temperature (21–25 °C), the humidity of 50–60%, and photoperiod of 12 h. Three rats were housed in a cage and had free access to standard food (American Institute of Nutrition Rodent Diets-93G (AIN-93G diet) and water. All experimental procedures were approved by the Peking University Animal Research Committee, following the Guide for the Care and Use of Laboratory Animals (NIH publication no. 85-23, revised 1996).

After one week of acclimatization, seventy rats were randomly divided into seven groups (10/group): normal group, ethanol group, whey protein group (220 mg/kg body weight, as a protein reference), omeprazole group (20 mg/kg body weight, as a reference of the anti-ulcer drug) [4,5,24], and three WOPs intervention groups at different doses (220, 440, 880 mg/kg body weight, namely WOPs-LG, WOPs-MG, WOPs-HG respectively). Rats in whey protein, omeprazole, and WOPs groups were orally gavage with distilled water as a vehicle, while normal group and ethanol group were given vehicle alone once a day for 30 consecutive days. The body weight and food consumption of rats were recorded by an electronic weighing scale once a week. 

At day 30, after the final administration, all rats were fasted for 24 h but given free access to water. Then rats were gavage with absolute ethanol at 5 mL/kg body weight to induce gastric ulceration based on reference to other research and pre-experiment [8,25,26,27,28]. One hour later, animals were sacrificed, a blood sample was collected from the femoral artery and then centrifuged at 3000 rpm for 15 min to obtain serum and preserved at −80 °C; the stomach was immediately removed, and the gastric juice was collected into a tube and then the pH of gastric content was recorded with a digital pH meter.

### 2.4. Microscopic Evaluation of the Gastric Lesions

The stomach was opened along the greater curvature, washed clean with cold saline, and blotted dry with filter paper, then the gross lesions of gastric tissue were observed and evaluated. The gastric ulcer index (GUI) was scored according to the Guth standard with some modifications [8,29,30], as shown in Table 1.

### 2.5. Biochemical Assays and Enzyme-Linked Immunobsorbent Assay

The levels of serum ALT and AST were determined by an automatic biochemical analyzer (Olympus Corporation, Tokyo, Japan), the gastric tissue homogenate of different treated groups were used to estimate the PG1, PG2, PGE2, mucin contents, inflammatory parameters (MPO, TNF-α, IL-6, IL-1β, IL-10), oxidative stress biomarkers (NO, SOD, CAT, GSH, MDA) by ELISA kits according to the protocol provided by the manufacturer.

### 2.6. Western Blot Analysis

The expression of Bax, caspase-3, Bcl-2, HSP70, NF-κB (p65), and IκBα proteins in gastric tissue of experiment rats was determined by Western blot analysis according to the previously described procedures with some optimizing modifications [31,32]. In brief, total proteins were extracted from homogenates using RIPA lysis buffer and the concentration was determined according to the BCA method. Then, 20 ug sample was electrophoresed through sodium dodecyl sulfate-polyacrylamide gel (10%) electrophoresis and electrotransferred to polyvinylidene fluoride membranes (Millipore, Billerica, MA, USA) for one hour. Next, the membranes were incubated with 5% (*m*/*v*) skimmed milk for four hours at room temperature, followed by incubation with primary antibodies against β-actin at 1:2000 (loading control), NF-κB at 1:1000, IκB at 1:5000, HSP 70 at 1:1000, Bax at1:1000, Bcl-2 at 1:2000, and caspase-3 at 1:2000 overnight at 4 °C. Then, the membranes were incubated with secondary antibody goat anti-rabbit IgG (1:10000) at room temperature for four hours. Finally, protein bands were visualized and detected by the chemiluminescence reaction and Hyperfilm ECL, then quantified and processed by Image-Pro Plus (IPP) software.

### 2.7. Statistical Analysis

All values were presented as means ± standard deviations (SDs). Variances in the measurement data were checked for homogeneity by Bartlett’s test. When the data were homogeneous, the one-way analysis of variance test with least significant difference (LSD) or Dunnett’s T3 methods was used to measure the significance of differences among groups. The levels of *p* < 0.05 was considered to be statistically significant.

## 3. Results

### 3.1. Effect of WOPs on Body Weight and Food intake

During the 30 days of the experiment, it was observed that daily intervention of WOPs did not cause any signs of toxicity or mortality in rats, and there was no significant difference in the bodyweight among all groups (*p* > 0.05). The total food intake was significantly higher in WOPs-HG groups than that in the normal control, ethanol, and whey protein group (*p* < 0.05), but no significant change in food utilization was observed between groups (*p* > 0.05) (Table 2).

### 3.2. Effect of WOPs on Gross Evaluation and Gastric Ulcer Index (GUI) of the Gastric Mucosa in Rats

**Figure 1:**
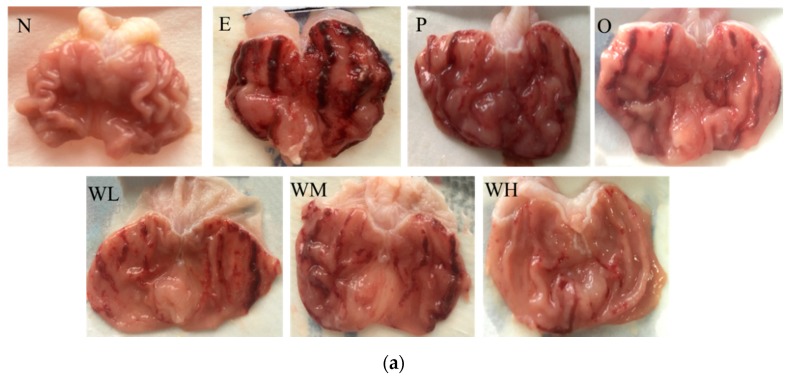

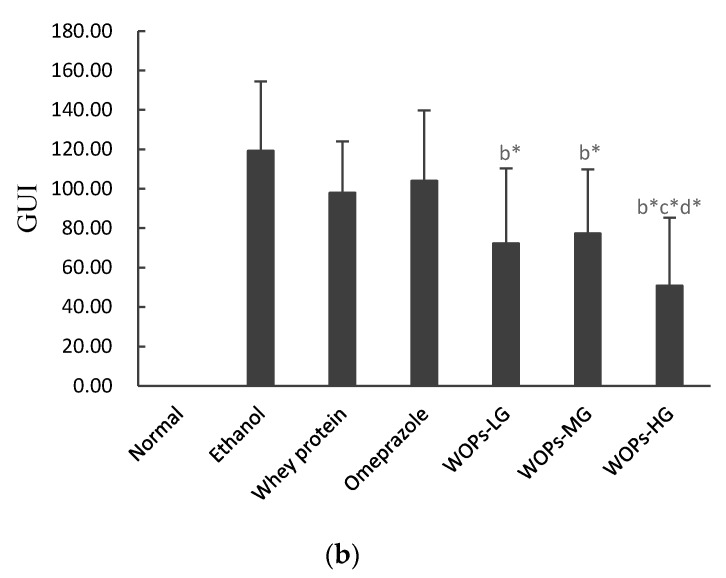
Effect of walnut oligopeptides (WOPs) pretreatment on gross evaluation (**a**) and gastric ulcer index (**b**) of gastric mucosa injury in ethanol-treated rats. N, normal control group; E, ethanol group; P, whey protein group (220 mg/kg body weight); O, omeprazole group (20 mg/kg body weight); WL, WOPs-LG (220 mg/kg body weight); WM, WOPs-LG (440 mg/kg body weight); WH, WOPs-HG (880 mg/kg body weight). Data were presented as mean ± SD (*n* = 10); b, c, d stand for in comparison with ethanol group, whey protein group and omeprazole group, respectively; * *p* < 0.05 was considered to have statistical differences. GUI, Gastric ulcer index; WOPs-LG, 220 mg/kg of walnut oligopeptides group; WOPs-MG, 440 mg/kg of walnut oligopeptides group; WOPs-HG, 880 mg/kg of walnut oligopeptides group.

As shown in Figure 1a, no visible lesion exhibited in the stomach of rats in the normal group, the surface of the stomach was pink, and the gastric mucosa was smooth, intact, and regular (group N). Rats in six groups administrated with absolute ethanol at 5 mL/kg displayed varying degrees of hemorrhagic gastric lesions and the incidence rate of injury was 100%, indicating that ethanol has caused gastric mucosa injury successfully (group E~WH). Rats in ethanol group suffered the most severe gastric mucosal damage with a wide range of lesions appearing as extended hemorrhagic bands (group E), while the whey protein group (group P) and omeprazole group (group O) revealed mild damage compared with ethanol group. However, WOPs administration groups (group WL, WM and WH) exhibited moderate to slight gastric mucosa injuries in comparison with ethanol group, and among three WOPs groups, the gastric mucosa injuries of rats in the WOPs-HG (880 mg/kg body weight) group were the least severe and remarkably better than in whey protein group and omeprazole group.

The results presented in Figure 1b illustrated that the gastric ulcer index (GUI) substantially increased of rats in the ethanol group. The administration with WOPs significantly reduced GUI in comparison with the ethanol group (*p* < 0.05). Gastric mucosa of rats in the WOPs-HG (880 mg/kg body weight) was mainly spotted hemorrhage, its GUI was distinctly lower than that of whey protein group and the omeprazole group (*p* < 0.05). However, the GUI of rats in the omeprazole group and whey protein group was lower than that of the ethanol group, but the difference was not significant (*p* > 0.05). The intervention dose of omeprazole positive control group was converted according to the recommended dosage to body weight ratio according to the drug instructions [4].

### 3.3. Effect of WOPs on Gastric Content pH, Pepsinogen, Gastric Mucin Content and Biochemical Analysis

As shown by the results in Table 3, oral administration of absolute ethanol significantly increased the gastric content pH, pepsinogen (includes PG1 and PG2) levels, and decreased gastric mucus contents in comparison with the normal rats (*p* < 0.05); while animals administrated with WOPs or whey protein or omeprazole groups were significantly decreased PG1, PG2 levels and increased mucin contents compared with ethanol group, and gastric content pH in WOPs-HG (880 mg/kg body weight) group was lower than that in ethanol group (*p* < 0.05). Besides, the activity of PG1 in WOPs intervention group was higher, while PG2 was lower than that in whey protein group (*p* < 0.05); and in comparison with rats in WOPs intervention groups, the gastric contents pH and PG2 level in omeprazole group were significantly increased, while PG1 level and PGR (ratio of PG1 to PG2) were decreased (*p* < 0.05). On the other hand, the ethanol group exhibited a significant increase in serum AST and ALT levels compared with untreated rats (*p* < 0.05). However, animals administrated with WOPs significantly reduced the elevated of ALT in three WOPs groups and AST in WOPs-HG group (*p* < 0.05).

### 3.4. Effect of WOPs on PGE2, NO, and MPO Levels in Gastric Tissue of Rats

**Figure 2:**
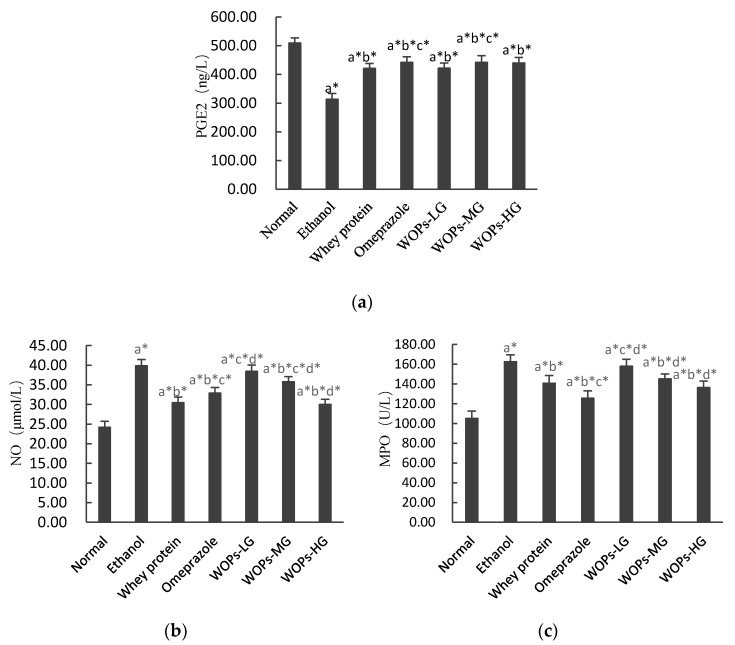
Effects of WOPs on gastric PGE2 (**a**), NO (**b**), and MPO (**c**) levels in ethanol-induced rats. Values were expressed as mean ± SD (*n* = 10); a, b, c, d means compared with normal group, ethanol group, whey protein group, and omeprazole group separately; * *p* < 0.05 was considered to have statistical differences. PGE2, prostaglandin E2; NO, nitric oxide; MPO, myeloperoxidase. WOPs-LG, 220 mg/kg of walnut oligopeptides group; WOPs-MG, 440 mg/kg of walnut oligopeptides group; WOPs-HG, 880 mg/kg of walnut oligopeptides group.

As shown in Figure 2, ethanol administration significantly reduced the content of PGE2, elevated levels of NO and MPO when compared with rats in the normal group (*p* < 0.05). The pretreatment of whey protein, omeprazole or WOPs significantly increased PGE2 content, decreased NO and MPO levels compared with ethanol group (*p* < 0.05). In comparison with whey protein group, the levels of PGE2 in WOPs-MG, NO in WOPs-LG and WOPs-MG, MPO in WOPs-LG were slightly higher (*p* < 0.05); whereas PGE2 in WOPs-LG and WOPs-HG, NO in WOPs-HG, MPO in WOPs-MG and WOPs-HG show no significant difference (*p* > 0.05). In addition, NO and MPO levels in three WOPs intervention groups were higher than those in the omeprazole group (*p* < 0.05), but there was no significant difference in PGE2 level (*p* > 0.05).

### 3.5. Effect of WOPs on Ethanol-Induced Oxidative Stress in Gastric Tissue of Rats

**Figure 3:**
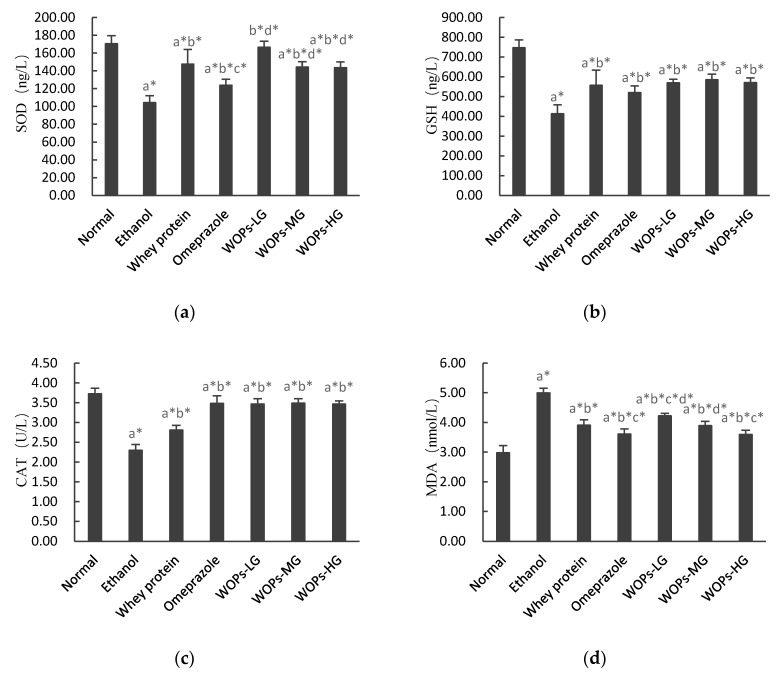
Effects of WOPs on SOD (**a**), GSH (**b**), CAT (**c**), MDA (**d**) levels in ethanol-induced rats. Values are presented as mean ± SD (*n* = 10); a, b, c, d means compared with normal group, ethanol group, whey protein group, and omeprazole group separately; * *p* < 0.05 was considered to have statistical differences. SOD, superoxide dismutase; GSH, reduced glutathione; CAT, Catalase; MDA, malondialdehyde; WOPs-LG, 220 mg/kg of walnut oligopeptides group; WOPs-MG, 440 mg/kg of walnut oligopeptides group; WOPs-HG, 880 mg/kg of walnut oligopeptides group.

As shown in Figure 3, the results indicated rats administrated with 5 mL/kg absolute ethanol showed decreased SOD, GSH, and CAT levels, whereas their gastric mucosal malondialdehyde (MDA) levels were significantly elevated compared with rats in the normal group (*p* < 0.05). Whey protein, omeprazole, and WOPs-treated rats significantly reversed those changes compared with the ethanol group (*p* < 0.05). Furthermore, MDA levels of rats in t WOPs-HG were significantly reduced compared with whey protein group (*p* < 0.05), SOD levels of rats in three WOPs intervention groups were increased compared with omeprazole group (*p* < 0.05). However, there was no significant change in GSH and CAT levels between WOPs groups and whey protein group or omeprazole group (*p* > 0.05).

### 3.6. Effect of WOPs on Inflammatory Cytokines in Gastric Tissue of Rats

**Figure 4:**
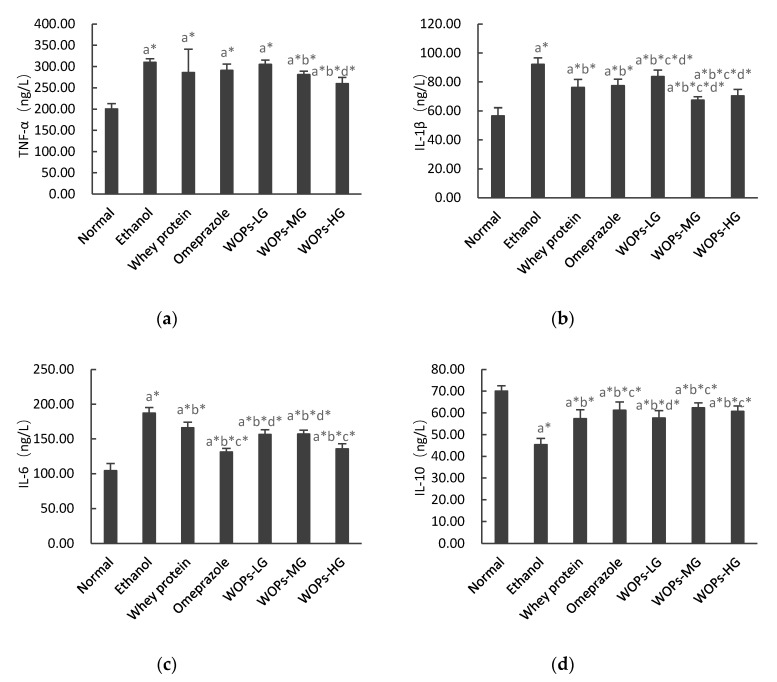
Effects of WOPs on TNF-α (**a**), IL-1β (**b**), IL-6 (**c**), and IL-10 (**d**) levels in ethanol-induced rats. Data are expressed as mean ± SD (*n* = 10); a, b, c, d represent compared with normal group, ethanol group, whey protein group, and omeprazole group, respectively; * *p* < 0.05 was considered to have statistical differences. Tumor necrosis factor α, TNF-α; interleukin (IL)-6, IL-6; interleukin (IL)-1β, IL-1β; IL-10, interleukin (IL)-10; WOPs-LG, 220 mg/kg of walnut oligopeptides group; WOPs-MG, 440 mg/kg of walnut oligopeptides group; WOPs-HG, 880 mg/kg of walnut oligopeptides group.

As shown in Figure 4, the levels of TNF-α, IL-6, and IL-1β after ethanol exposure were markedly higher, while IL-10 was significantly decreased compared to the rats of normal group (*p* < 0.05), which indicated that ethanol-induced inflammatory response generated multiple cytokines in rats. WOPs pretreatment greatly suppressed the increased TNF-α, IL-6, IL-1β and IL-10 levels in gastric tissue of rats (*p* < 0.05), except TNF-α in WOPs-LG group, which is similar to the difference between whey protein, omeprazole groups, or ethanol group. Moreover, the levels of IL-1β in WOPs-MG and WOPs-HG, IL-6 in WOPs-HG groups were lower, while IL-1β in WOPs-LG, IL-10 in the WOPs-MG and WOPs-HG groups were higher than those in rats of whey protein group (*p* < 0.05); but no significant difference was observed in TNF-α between WOPs groups and whey protein group (*p* > 0.05). Compared with omeprazole group, TNF-α in WOPs-HG group, IL-1β in WOPs-MG and WOPs-HG groups, and IL-10 in the WOPs-LG group were distinctly decreased, while IL-6 in WOPs-LG, WOPs-MG groups were greatly increased (*p* < 0.05). 

### 3.7. Effect of WOPs on the NF-κB p65, IκBα, HSP70, Bcl-2, Bax and Caspase-3 Expression in Gastric Tissue

The protein expression of nuclear factor-κB p65 (NF-κB p65), inhibitor kappa Bα (IκBα), heat shock protein70 (HSP70), Bcl-2, Bax, and caspase-3 in the gastric tissue were examined by Western blot analysis. 

**Figure 5:**
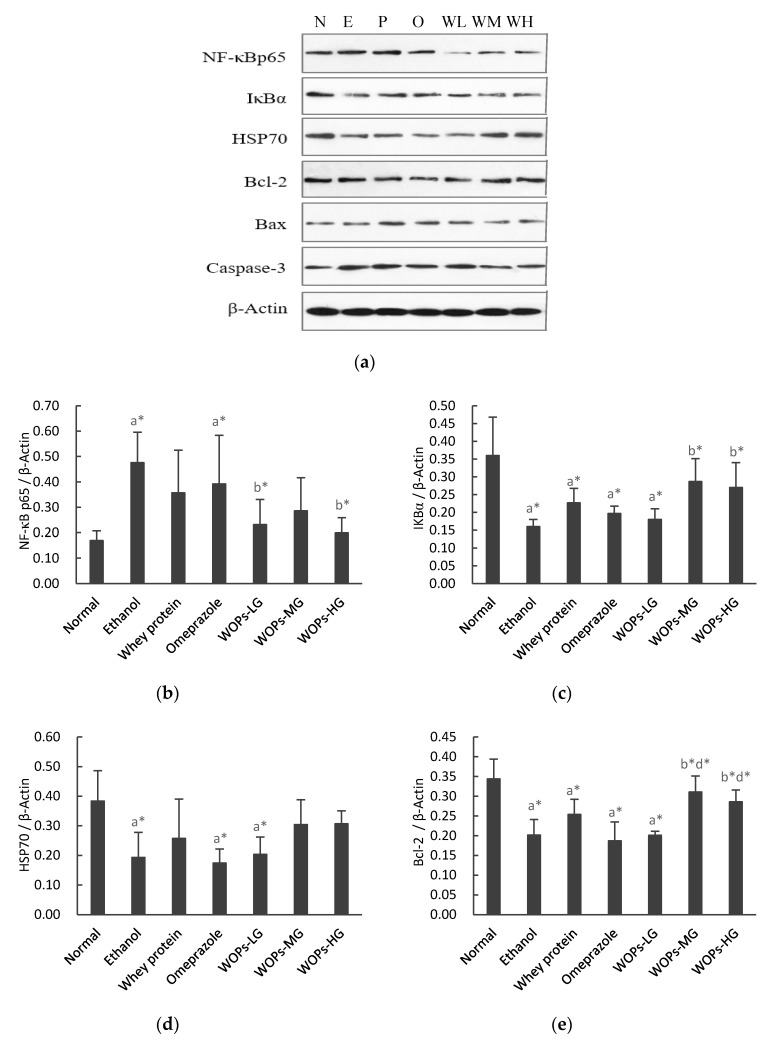

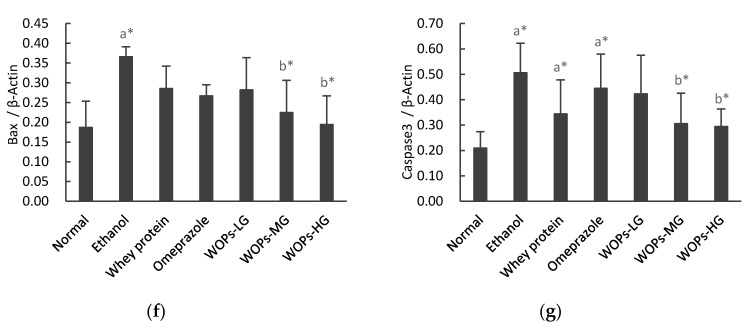
Effect of WOPs on the apoptosis-related protein expression in rats. (**a**) Apoptosis-related protein levels were analyzed by Western blot. N, normal control group; E, ethanol group; P, whey protein group (220 mg/kg body weight); O, omeprazole group (20 mg/kg body weight); WL, WOPs-LG (220 mg/kg body weight); WM, WOPs-LG (440 mg/kg body weight); WH, WOPs-HG (880 mg/kg body weight). (**b**) Effect of WOPs on the expression of NF-κB p65 in gastric tissue of rats. (**c**) Expression of IκBα in gastric tissue of rats. (**d**) Expression of HSP70 in gastric tissue of rats. (**e**) Expression of Bcl-2 in gastric tissue of rats. (**f**) Expression of Bax in gastric tissue of rats. (**g**) Expression of Caspase-3 in gastric tissue of rats. Data are expressed as mean ± SD (*n* = 10); a, b, d represent compared with the normal group, ethanol group, and omeprazole group separately; * *p* < 0.05 was considered to have statistical differences. NF-κB p65, nuclear factor-κB p65; IκBα, inhibitor kappa Bα; WOPs-LG, 220 mg/kg of walnut oligopeptides group; WOPs-MG, 440 mg/kg of walnut oligopeptides group; WOPs-HG, 880 mg/kg of walnut oligopeptides group.

As shown in Figure 5b,c, compared with the normal group, the expression of NF-κB p65 significantly increased and the expression of IκBα reduced in ethanol group (*p* < 0.05). Whereas the expression of NF-κB p65 in WOPs-LG and WOPs-HG groups was significantly down-regulated, IκBα in WOPs-MG and WOPs-HG groups was significantly up-regulated in comparison with those in ethanol group (*p* < 0.05), and there was no significant difference between WOPs group, whey protein group, and omeprazole group (*p* > 0.05).

The data illustrated in Figure 5d indicated that ethanol administration resulted in significant down-regulation of HSP 70 expression of animals in ethanol group, omeprazole group, and WOPs-LG group compared with the normal group (*p* < 0.05). However, no statistical difference was observed among other groups (*p* > 0.05).

The data presented in Figure 5e–g showed that the gastric mucosa of rats in ethanol group had significant apoptosis damage, which was exhibited by the down-regulation of anti-apoptosis protein Bcl-2 and up-regulation of the pro-apoptotic proteins Bax and caspase-3 (*p* < 0.05), while no significant difference was observed in whey protein group or omeprazole group in comparison with those in ethanol group (*p* > 0.05). Nevertheless, the expression of Bcl-2 was significantly up-regulated, while Bax and caspase-3 were markedly down-regulated of rats in WOPs-MG and WOPs-HG groups when compared with the ethanol group (*p* < 0.05), which indicated that WOPs intervention can alleviate the gastric mucosal apoptosis induced by ethanol to a certain extent. Besides, the expression of Bcl-2 in WOPs-MG and WOPs-HG groups were higher than those of omeprazole group (*p* < 0.05), whereas the expression of Bax and caspase-3 between WOPs groups and omeprazole group did not differ significantly (*p* > 0.05).

## 4. Discussion

Nowadays, natural products, especially food-derived peptides, have gradually attracted researchers’ attention because of its varied biological effects with reduced side effects [33,34]. WOPs, which are mainly composed of small-molecule oligopeptides derived from walnuts (*Juglans regia* L.), have been reported to have various physiological functions such as anti-oxidants, anti-inflammatory, anti-fatigue, and attenuating irradiation-induced hematopoietic [22,23]. However, the effects of WOPs on ethanol-induced gastric mucosal injury has not been reported. Thus, we aimed to treat the Sprague–Dawley (SD) rats with walnut (*Juglans regia* L.) oligopeptides (WOPs) by oral gavage to investigate the gastric protective effect of WOPs on gastric mucosal injury induced by absolute ethanol (5 mL/kg). Whey protein and omeprazole were used as reference groups. Whey protein is composed of several protein fractions including β-lactoglobulin, α-lactalbumin, and immunoglobulin, and has a role in a variety of physiological activities, such as enhance immunity, antioxidation, improve learning and memory abilities, and promote growth [12]. Omeprazole is a proton pump inhibitor (PPI), which is widely used in the short-term and long-term treatment of peptic ulcer disease attribute to the effect in causing a relatively complete suppression of acid secretion [5]. Most studies of ethanol-induced gastric mucosal damage have been performed in rodent models, but there is no generally accepted gavage dose of alcohol to date. According to multiple pieces of literature, absolute ethanol administered at a dose of 5 mL/kg in rodents was used more frequently, and the gastric mucosa injury can occur about 30 min and generally reach a peak 60 min after alcohol intake, which took into account differences in alcohol metabolism between rodents and humans [8,25,26,27,28]. In pre-experiments, we found that the dose of absolute ethanol caused obvious damage to gastric mucosa with low mortality of rats. Therefore, we developed the gastric mucosa injury model in SD rats by gavage 5 mL/kg absolute ethanol and then rats were sacrificed one hour later in this study. The results showed that rats in ethanol group suffered severe gastric mucosal damage, exhibited edema, the mucosal surface was dark red, mucosal erosion, punctate, linear, cord-like and even flaky hemorrhage (Figure 1), and the incidence rate of injury was 100%, indicating that ethanol has caused gastric mucosa injury successfully, which is in accordance with previous studies [8,30]. However, gross observation showed that whey protein, omeprazole, and three WOPs groups had less gastric lesions and lower GUI values in comparison with the ethanol group, and the gastric mucosa injuries of rats in the WOPs-HG group was the lightest and significant and better than that of whey protein group or omeprazole group. The results demonstrated that animal pre-treated with WOPs, especially at a dose of 880 mg/kg body weight, could considerably reduce the gastric mucosal hemorrhage injury caused by ethanol.

Gastric mucosal injury is a multifactorial pathological process that involved endogenous and exogenous factors, its basic pathophysiology mechanism includes imbalance between some pro-inflammation factors (including hydrochloric acid, pepsin, and reactive oxygen species) and defensive factors (such as mucus-bicarbonate barrier, mucosal blood flow, and some cytokines) [35,36,37,38]. Although the complex pathological mechanism of ethanol-induced gastric mucosal damage, the interaction of gastric acid and pepsin is still considered to be the major cause of gastric mucosal injury [39]. Excessive secretion of gastric acid would cause weakened barrier function, resulting in self-digestion of gastric mucosa tissue and inducing the occurrence of the ulcer [40,41]. Pepsin is transformed from pepsinogen (PG) secreted by main cells through activation of hydrogen ions [42]. PG1 and PG2 levels are important indicators of gastric mucosal injury, mirroring the morphology and functional status of gastric mucosa, while the reduction of PGR (the ratio of PG I to PG II) can be used to examine the progress of atrophic gastritis [43]. The results of this experiment illustrated that acute destruction of ethanol induced a remarkable elevation in gastric content pH and the levels of PG1, PG2 in ethanol group; while the pH of gastric contents in omeprazole group were higher than other groups. However, the administration with WOPs reversed these changes to some extent compared with the ethanol group. In addition, mucin is a critical glycoprotein element that forms a protective mucus layer on the gastric mucosa [30]. The mechanism of ethanol-induced depletion of gastric mucin is mainly that acetaldehyde directly inhibits the galactosyltransferase activity, which is a vital component in mucin synthesis, thus affecting the synthesis of ethanol dehydrogenase and mucin glycoprotein [5]. In the present study, the result demonstrated that the contents of mucin of rats in WOPs intervention groups were distinctly higher than that of rats in the ethanol group. Moreover, the levels of serum alanine aminotransferase (ALT) and aspartate aminotransferase (AST) have been regarded as sensitive pathological markers of ethanol-induced liver dysfunction. WOPs could prevent the elevated ALT, AST levels of rats in the ethanol group, which suggested that WOPs exerted a certain protective effect on impaired liver function caused by absolute ethanol in the present study.

With any etiology, various factors are involved in the pathological process of gastric mucosa injury, such as prostaglandin E2 (PGE2), nitric oxide (NO), myeloperoxidase (MPO), heat shock proteins (HSP70), etc., [39]. Prostaglandins, in particular prostaglandin E2 (PGE2), are the major defensive factors against most gastric mucosa irritation induced by various risk factors. PGE2 can primarily protect gastric mucosal by stimulating gastric mucus and bicarbonate secretion, increasing the mucosal blood flow, suppressing the aggregation of leukocyte, and attenuating cytotoxic damage by participating in various mechanisms [5,39,44]. The present study demonstrated that compared with normal rats, the gastric mucosa PGE2 levels in the ethanol group rats were significantly reduced (Figure 2a), suggesting that ethanol causes the inactivation of prostaglandin synthase, which leads to a decrease in prostaglandin biosynthesis, which is in accordance with other related researches [4,5,8,30]. However, WOPs remarkably enhanced the level of PGE 2 in comparison with ethanol group, indicating that PGE2 participated in the defense mechanism of WOPs against ethanol-induced gastric mucosal injury. Additionally, neutrophils are a considerable source of inflammatory cytokines and can induce reactive oxygen that can result in gastric mucosal damage. Previous studies have found that as a crucial indicator of neutrophil infiltration, an increase in MPO level was often observed in the gastric ulcer caused by ethanol [5,30]. Nitric oxide synthase (NOS) can be divided into two categories, the constructive nitric oxide synthase (cNOS) and inducible nitric oxide synthase (iNOS). cNOS can constantly release NO at low levels under physiological situations [45]. Whereas, iNOS can produce a large amount of inducible NO after being activated by endotoxin or inflammatory cytokines, causing vascular microcirculation disturbance and gastric mucosa injury [42,46,47]. In this study, the MPO and NO levels were considerably elevated in rats in the ethanol group when compared with those of the normal group (Figure 2b,c). However, the considerable upsurge of MPO and NO levels were markedly suppressed by treatment with WOPs, whey protein, and omeprazole compared with the ethanol group rats. Besides, the results showed that omeprazole has a higher ability to inhibit MPO and NO levels than WOPs, but no distinct difference was observed between whey protein and WOPs. Heat shock protein70 (HSP70) has a protective effect against ethanol-induced gastric mucosal injury [2,48]. However, no significant difference was observed between WOPs groups and ethanol group in this study (Figure 4d). The above results suggested that WOPs had a protective effect on gastric mucosa, enhanced PGE2 activity in gastric mucosal, and caused a reduction of MPO and NO levels in the ulcerated tissue.

Oxidative stress has been suggested to be involved in the pathogenesis of gastric mucosal damage caused by ethanol, which is due to the imbalance between the substantial production of reactive oxygen species (ROS) and rapid depletion of endogenous antioxidant capacity [6,49]. A chief source of oxygen free radicals generation could be ascribed to the infiltration of neutrophils, and it is closely related to the imbalance of cellular homeostasis and the excessive production of lipid peroxides [26]. The impairment of antioxidant defense mechanisms was induced by ethanol basically through enhancing the generation of cellular oxidants (e.g., O2•−) and lipid peroxidation products (e.g., MDA) and depleting the number of antioxidants (e.g., SOD, GSH, CAT) in the gastric mucosa, which in turn causes mitochondrial membrane changes in permeability and depolarization, and eventually led to cell death and apoptosis [5,50]. Therefore, increased activity of scavenging enzymes is an effective way to alleviate oxidative damage, which plays a major defensive role in the genesis of ethanol-induced gastric ulcers induced by ethanol [4,48,51]. Meanwhile, malondialdehyde (MDA) is the final production of lipid peroxidation and is a key marker to estimate indirectly the level of lipid peroxidation [5,35]. Our project outcomes indicated that administration with of 5 mL/kg absolute ethanol reduced the levels of SOD, CAT, and GSH, while it elevated the MDA levels in ethanol-administered rats. However, administration with WOPs distinctly altered the reduction of those antioxidant enzymes and the increase of MDA levels. Furthermore, the intervention of WOPs markedly enhanced SOD levels compared with omeprazole group and attenuated MDA levels compared with the whey protein group. These results suggested that WOPs have the powerful ability to improve absolute ethanol-induced oxidative stress imbalance of gastric mucosal.

Inflammation is a pivotal pathological reaction of ethanol-related peptic ulcer, which is mainly characterized by increased secretion of diverse proinflammatory factors, such as TNF-α, IL-6, and IL-1β, and inhibition of the production of anti-inflammatory cytokines such as IL-10 [7,52]. TNF-α is relevant to the inflammatory reaction, lipid metabolism, and apoptotic injuries in the development of gastric ulcer [48]. In addition, this pro-inflammatory cytokine can promptly trigger the expression of the transcription factor nuclear factor κB (NF-κB), which in turn activates the release of various cytokines, thus aggravating gastric mucosa injury [30,48]. Moreover, IL-10 is mainly produced by Th2 cells or mononuclear macrophages, is a multifunctional negative regulatory factor that plays a down-regulating role in an inflammatory response, which can inhibit and antagonize the production and activity of pro-inflammatory factors. Consequently, inhibiting the increase of inflammatory cytokines is essential to alleviate gastric mucosal injury caused by ethanol. In this study, the levels of TNF-α, IL-6, IL-1β in ethanol group were significantly elevated, while the levels of IL-10 were decreased when compared with those of rats in the normal group, indicating the involvement of inflammatory response in ethanol-induced gastric mucosa injury, which was in accordance with other similar reports [26,30,53]. Notably, the administration with WOPs remarkably improved the levels of these inflammatory cytokines in comparison with rats in the ethanol group. These results indicate that WOPs-treated rats displayed a remarkable attenuation of ethanol-induced inflammation reaction via suppressing TNF-α, IL-6, IL-1β and increasing IL-10 and MPO (Figure 2c) levels of gastric mucosal.

Previous studies have shown that apoptosis is closely related to the occurrence of gastric mucosal damage, and excessive apoptosis will destroy the integrity of the mucosa and eventually induce gastric ulcer [8,54]. The cellular apoptosis caused by ethanol could be primarily attributed to the upregulation of pro-apoptosis protein Bax and caspase-3, and down-regulation of the anti-apoptosis protein Bcl-2, thereby resulting in gastric mucosa dysfunction [11,30]. Additionally, NF-κB is the predominant mediator of redox imbalance and excessive generation of inflammatory cytokines [8,48]. Generally, NF-κB interacts with inhibitor kappa B (IκB) to make it inactive in the cytoplasm under physiological conditions. When various stimuli lead it to return to phosphor-IκB, then IκB degrades, thereby translocating NF-kB to the nucleus and inducing the expression of its target genes, such as TNF-α and IL-1β [31,55,56]. The results demonstrated that the protein expression of NF-κB p65 (Figure 5b), Bax (Figure 5f), and caspase 3 (Figure 5g) were up-regulated, while the IκBα (Figure 5c) and Bcl 2 (Figure 5e) expression were down-regulated in rats in the ethanol group when compared with those of rats in the normal group. However, no significant difference was found in the whey protein group or omeprazole group when compared with the ethanol group. Notably, administration with WOPs reduced the expression of NF-κB p65, Bax, and caspase 3, and increased the expression of IκBα and Bcl 2 in comparison with those of ethanol rats, which was consistent with previously reported studies, showing WOPs could inhibit IR-induced splenocyte apoptosis and inflammation [23]. Moreover, comparing the gastroprotective effects of WOPs and omeprazole or whey protein, WOPs were superior in the regulation of anti-apoptosis protein Bcl-2 in comparison with omeprazole group, but there was no significant difference between both groups on NF-κB p65, Bax, and caspase-3 expression. Taken together, these results indicate that WOPs-treated rats exhibited a marked gastroprotective effect, primarily manifested by anti-inflammatory, anti-oxidative, and anti-apoptotic mechanisms, which could be ascribed to the resultant changes in gastric PGE2 levels and regulation of IκBα/NF-κB signal pathway in rats.

## 5. Conclusions

The above research results suggests that WOPs have an obvious protective effect on gastric mucosal injury caused by ethanol. The gastroprotective activity of WOPs is primarily because of its effect on attenuating ethanol-induced gastric mucosal hemorrhagic injury, reducing gastric ulcer index, enhancing PGE2 and oxidative stress parameters, suppressing MPO, proinflammatory factors, and lipid peroxidation indicators changed by oral gavage with 5 mL/kg ethanol to rats, thereby mitigating gastric mucosa damage caused by ethanol. In addition, administration with WOPs could up-regulate the expression of anti-apoptosis protein (Bcl-2) and down-regulated the expression of pro-apoptotic proteins (Bax and caspase-3), and it might be associated with the alleviation of the NF-κB signaling pathway. Furthermore, we found that the optimal dose of WOPs supplementation in rats may be 880 mg/kg body weight, which showed better protective efficacy against ethanol-induced gastric mucosal damage, but further experimental verification is needed. This study first illustrated the gastroprotective effect of WOPs and provides an important prospect for the application of WOPs on ethanol-induced gastric mucosa injury. Further research is required to evaluate the protective effect of WOPs in a clinical setup and ascertained the optimal dose of WOPs supplementation in humans.

## Figures and Tables

**Table 1:** The scoring criteria of gastric ulcer index (GUI).

Severity of Erosion	1 Point	2 Points	3 Points	4 Points		
Spot erosion		-	-	-		
Erosion length	<1 mm	1–2 mm	2–3 mm	3–4 mm	>4 mm, segmented scored	
Erosion width	>2 mm, score doubled					
Total score	A sum of partial scores					

**Table 2:** Effects of walnut oligopeptides (WOPs) on body weight and food intake.

Groups	Body Weight (g)		Food Intake (g)	Food Utilization (%)
	Initial	Final		
Normal	218.63 ± 9.79	400.00 ± 47.56	712.82 ± 46.74	25.37 ± 5.46
Ethanol	218.23 ± 10.19	396.17 ± 31.58	733.38 ± 81.68	25.28 ± 4.31
Whey protein	218.88 ± 10.13	412.29 ± 49.08	722.21 ± 52.95	26.68 ± 5.87
Omeprazole	218.60 ± 12.51	403.45 ± 46.70	750.68 ± 40.59	24.75 ± 5.37
WOPs-LG	221.75 ± 6.33	400.40 ± 46.15	748.74 ± 82.38	24.54 ± 4.12
WOPs-MG	220.14 ± 8.58	398.68 ± 33.54	739.33 ± 51.50	25.52 ± 2.43
WOPs-HG	221.15 ± 7.06	425.73 ± 55.07	787.00 ± 64.67 ^a^*^b^*^c^*	27.08 ± 3.32

Food utilization = (Final body weight − initial body weight)/food intake × 100%. Values were presented as mean ± SD (*n* = 10), a* means compared with normal group: *p* < 0.05; b* means compared with ethanol group: *p* < 0.05; c* means compared with whey protein group: *p* < 0.05. WOPs-LG, 220 mg/kg of walnut oligopeptides group; WOPs-MG, 440 mg/kg of walnut oligopeptides group; WOPs-HG, 880 mg/kg of walnut oligopeptides group.

**Table 3:** Effect of WOPs on gastric content pH, pepsinogen, gastric mucin and serum ALT, AST levels.

Groups	pH	PG1 (μg/L)	PG2 (μg/L)	PGR	Mucin (ng/L)	ALT (U/L)	AST (U/L)
Normal	2.42 ± 0.77	5.09 ± 0.35	2.89 ± 0.13	1.77 ± 0.18	4.42 ± 0.23	60.83 ± 17.37	211.50 ± 57.15
Ethanol	5.15 ± 0.95 ^a^*	8.74 ± 0.43 ^a^*	4.90 ± 0.17 ^a^*	1.79 ± 0.08	2.47 ± 0.16 ^a^*	83.75 ± 15.74 ^a^*	300.80 ± 56.03 ^a^*
Whey protein	4.08 ± 1.52	6.10 ± 0.32 ^a^*^b^*	4.20 ± 0.24 ^a^*^b^*	1.46 ± 0.09 ^b^*	3.39 ± 0.14 ^a^*^b^*	68.11 ± 15.28 ^b^*	269.20 ± 58.54 ^a^*
Omeprazole	6.54 ± 1.17 ^a^*^b^*^c^*	5.81 ± 0.25 ^a^*^b^*	4.27 ± 0.21 ^a^*^b^*	1.36 ± 0.09 ^a^*^b^*	3.59 ± 0.17 ^a^*^b^*^c^*	71.00 ± 19.03	262.00 ± 44.47 ^a^*
WOPs-LG	4.16 ± 1.26 ^d^*	7.46 ± 0.18 ^a^*^b^*^c^*^d^*	4.40 ± 0.20 ^a^*^b^*^c^*	1.71 ± 0.09 ^c^*^d^*	3.41 ± 0.17 ^a^*^b^*	68.45 ± 8.70 ^b^*	274.78 ± 53.67 ^a^*
WOPs-MG	3.14 ± 1.58 ^d^*	6.83 ± 0.36 ^a^*^b^*^c^*^d^*	4.00 ± 0.18 ^a^*^b^*^c^*^d^*	1.71 ± 0.11 ^c^*^d^*	3.29 ± 0.15 ^a^*^b^*^d^*	66.50 ± 12.78 ^b^*	283.00 ± 33.36 ^a^*
WOPs-HG	2.75 ± 0.44 ^b^*^d^*	6.97 ± 0.35 ^a^*^b^*^c^*^d^*	3.87 ± 0.25 ^a^*^b^*^c^*^d^*	1.80 ± 0.16 ^c^*^d^*	3.22 ± 0.13 ^a^*^b^*^c^*^d^*	64.60 ± 10.16 ^b^*	247.38 ± 29.21 ^b^*

The PGR was calculated as the PG1 (μg/L) to PG2 (μg/L). Values were presented as mean ± SD (*n* = 10), a* means compared with normal group: *p* < 0.05; b* means compared with ethanol group: *p* < 0.05; c* means compared with whey protein group: *p* < 0.05; d* means compared with omeprazole group: *p* < 0.05. ALT, alanine aminotransferase; AST, aspartate aminotransferase; WOPs-LG, 220 mg/kg of walnut oligopeptides group; WOPs-MG, 440 mg/kg of walnut oligopeptides group; WOPs-HG, 880 mg/kg of walnut oligopeptides group.
